# A simple scoring system to predict early recurrence of Bismuth–Corlette type IV perihilar cholangiocarcinoma

**DOI:** 10.1093/gastro/goz012

**Published:** 2019-04-21

**Authors:** Ding-Zhong Peng, Jiong Lu, Bei Li, Hai-Jie Hu, Xi-Wen Ye, Xian-Ze Xiong, Nan-Sheng Cheng

**Affiliations:** Department of biliary surgery, West China Hospital of Sichuan University, Chengdu, Sichuan, P. R. China

**Keywords:** early recurrence, Bismuth–Corlette classification, perihilar cholangiocarcinoma

## Abstract

**Background:**

Early recurrence has been reported to be predictive of a poor prognosis for patients with perihilar cholangiocarcinoma (pCCA) after resection. The objective of our study was to construct a useful scoring system to predict early recurrence for Bismuth–Corlette type IV pCCA patients in clinic and to investigate the value of early recurrence in directing post-operative surveillance and adjuvant therapy.

**Methods:**

In total, 244 patients who underwent radical resection for type IV pCCA were included. Data on clinicopathological characteristics, perioperative details and survival outcomes were analyzed. Survival curves were generated using the Kaplan–Meier method. Univariate and multivariate logistic-regression models were used to identify factors associated with early recurrence.

**Results:**

Twenty-one months was defined as the cutoff point to distinguish between early and late recurrence. Univariate and multivariate analysis revealed that CA19-9 level >200 U/mL, R1 resection margin, higher N category and positive lymphovascular invasion were independent predictors of early recurrence. The scoring system was constructed accordingly. The early-recurrence rates of patients with scores of 0, 1, 2, 3, 4, and 5 were 23.9%, 38.7%, 60.0%, 78.6%, 83.4%, and 100%, respectively. Adjuvant therapy was significantly associated with higher overall survival rate for patients with early recurrence, but not for those with late recurrence. Patients in the early-recurrence group with scores ≥2 had better prognoses after adjuvant therapy.

**Conclusions:**

A simple scoring system using CA19-9 level, N category, resection margin and lymphovascular invasion status could predict early recurrence, and thus might direct post-operative surveillance and adjuvant therapy for patients with type IV pCCA.

## Introduction

Perihilar cholangiocarcinoma (pCCA) is a devastating malignancy of the bile duct. Being situated in a confined and crucial space, it is encircled by the liver parenchyma, hepatic artery, portal vein, peripheral nerve system and bile duct, and has a strong tendency to infiltrate the abovementioned organs and tissues [1]. Bismuth–Corlette type IV pCCA is a locally advanced neoplasm that infiltrates the secondary biliary radicals of the bilateral hepatic ducts. Radical surgery involving extended hepatectomy, caudate-lobe resection, lymphadenectomy, vascular resection and reconstruction is usually performed to achieve negative resection margins for type IV pCCA [2, 3]. However, the survival outcome remains unsatisfactory for pCCA patients after radical resection, with 5-year overall survival rates of 10%–40% [4–6].

A high rate of post-operative recurrence (44%–80%) is significantly predictive of impaired prognosis for pCCA patients after curative-intent surgery [7–10]. Several studies demonstrated that the early recurrence of hepatobiliary tumors might be due to metastasis from the primary malignancy [11–13]. Zhang *et al.* [9] reported that early recurrence after curative-intent surgery impaired overall survival in pCCA patients, and further revealed that factors such as lymph-node status (N category), margin status, differentiation and caudate-lobe resection were predictors of early recurrence. However, previous findings may not be completely applicable to type IV pCCA due to its locally advanced nature. Moreover, a scoring system is needed to predict early recurrence and, above all, to help post-operative decisions on surveillance and therapy. Thus, the over-arching aim of our study was to construct a clinically useful scoring system to predict early recurrence of type IV pCCA and then to validate its value for the guidance of post-operative surveillance and adjuvant therapy.

## Material and methods

### Patient selection

The clinical records of 244 consecutive patients receiving radical surgery for type IV pCCA at West China Hospital of Sichuan University (China) between 1998 and 2008 were collected and analyzed. Patients with intrahepatic bile duct carcinoma, gallbladder carcinoma infringing the hilum, macroscopic positive resection margin or pre-operative chemotherapy and radiotherapy and patients who died within 90 days after the surgery were excluded. The Ethics Committee of West China Hospital of Sichuan University approved this retrospective study; the need for informed consent was waived.

### Pre-operative workup

The standard pre-operative assessment consisted of medical history, physical examination, laboratory tests, and radiographic examinations such as contrast-enhanced ultrasound, contrast-enhanced computed tomography and/or magnetic resonance cholangiography. Pre-operative biliary drainage was performed in patients with obstructive jaundice (total bilirubin >85 μmol/L) by endoscopic nasobiliary drainage (ENBD) or percutaneous transhepatic cholangiodrainage (PTCD). Portal-vein embolism was performed 2–3 weeks before surgery for patients with future remnant liver volumes less than 40%.

### Treatments

According to pre-operative and intra-operative evaluation, different surgical strategies were selected, including extra-hepatic bile duct resection and caudate lobectomy combined with hemihepatectomy or trisegmentectomy. Patients routinely underwent resection of regional lymph nodes, such as the hilar, pericholedochal, periportal, common hepatic artery, and peripancreatic lymph nodes. Vascular resection and reconstruction were performed in patients with tumor-infiltrating vessels. After radical resection, post-operative concurrent chemoradiotherapy or chemotherapy was administered for all patients except those with both T1N0/T2N0-stage tumors and microscopic negative resection margins or those who refused further treatment. Patients received a total radiation dose of 40 Gy delivered as a split course of 20 Gy in 10 fractions (14 consecutive days in every 28 days as a cycle, for two cycles), followed by maintenance chemotherapy with 375 mg/m^2^ of 5-fluorouracil (5-FU) or 1000 mg/m^2^ of gemcitabine.

### Data collection

Details of the patients’ demographics, clinical examination, laboratory tests, radiological images, surgical procedures, and survival outcomes were collected. Presence of pCCA in resected tumor samples was determined during pathological examination. Tumor stage was determined on the basis of the 8th edition of the American Joint Committee on Cancer (AJCC) TNM staging system. Tumors with R0 resection (microscopically tumor-free margins) or R1 resection (microscopically positive margins) were defined as resectable, whereas those with R2 resection (macroscopically positive margins) were defined as unresectable. Post-operative complications were assessed with the Clavien–Dindo classification [14]. For those with more than one post-operative complication, the grade of severity was determined as the highest.

### Follow-up protocol

After discharge, all patients were routinely followed up every 3 months in the first year and every 6 months subsequently. Carbohydrate antigen 19-9 (CA19-9), carcinoembryonic antigen (CEA) and liver function were measured and hepatic ultrasonography was performed for surveillance. For those with suspected recurrence after curative resection, additional examinations, such as contrast-enhanced computed tomography and magnetic resonance imaging, were conducted for a definitive diagnosis. Overall survival was defined as the interval between the date of surgery and that of death, or from the date of surgery to the date of last observation for surviving patients.

### Statistical analysis

Data analysis was performed using SPSS 19.0 software (SPSS Inc., Chicago, IL, USA). Comparisons between two groups were performed using the *t*-test or the Wilcoxon test for continuous factors and the Chi-square test or Fisher’s exact test for categorical factors. Survival was estimated using the Kaplan–Meier method and the significance of differences in survival was determined by the log-rank test. To identify independent factors associated with early recurrence, parameters were examined with univariate and multivariate logistic-regression models. Two-tailed values of *P *<* *0.05 were considered statistically significant.

## Results

### Characteristics of the study population

The patient characteristics are shown in Table 1. In total, 244 patients (including 154 males and 90 females) underwent radical surgery for type IV pCCA. Curative resection included extra-hepatic bile duct resection and caudate lobectomy combined with left hemihepatectomy (*n *=* *128, 52.5%), right hemihepatectomy (*n *=* *86, 35.2%), extended left hemihepatectomy (*n *=* *7, 2.9%), extended right hemihepatectomy (*n *=* *6, 2.5%), left trisegmentectomy (*n *=* *11, 4.5%), and right trisegmentectomy (*n *=* *6, 2.5%). Regional lymph-node resection was routinely performed in all patients. Of the 217 patients with obstructive jaundice, 183 patients with total bilirubin levels above 85 μmol/L underwent pre-operative biliary drainage including ENBD (*n *=* *56) and PTCD (*n *=* *127). Thirteen patients underwent portal-vein embolism. A total of 136 patients underwent adjuvant therapy; of them, 50 (36.8%) received chemotherapy alone, 83 (61.0%) received chemoradiotherapy, and 3 (2.2%) received radiotherapy alone.

**Table 1.** Characteristics of 244 patients with perihilar cholangiocarcinoma

Variable	Value
Median age, years (range)	61 (26–82)
Sex, *n* (%)	
Male	154 (63.1%)
Female	90 (36.9%)
Hypertension, *n* (%)	95 (38.9%)
Diabetes, *n* (%)	35 (14.3%)
Chronic hepatobiliary disease, *n* (%)	
Alcoholic liver disease	3 (1.2%)
Nonspecific cirrhosis	3 (1.2%)
Primary sclerosing cholangitis	1 (0.4%)
Cholecystolithiasis	36 (14.8%)
Choledocholithiasis/hepatolithiasis	57 (23.4%)
BMI, mean (range)	22.0 (17.2–28.6)
ASA score, *n* (%)	
1	6 (2.5%)
2	133 (54.5%)
3	105 (43.0%)
Total bilirubin, μmol/L (mean ± SD)	209.41 ± 174.40
CA19-9, U/mL, (mean ± SD)	403.53 ± 368.75
Albumin, g/L, (mean ± SD)	37.02 ± 5.24
Total lymph nodes evaluated, median (range)	3 (1–9)
Positive lymph-node number, median (range)	0 (0–7)
Median operative time, min (range)	250 (110–720)
Mean blood loss, mL (range)	600 (100–2000)
Blood transfusion, *n* (%)	80 (32.8%)
Any complications, *n* (%)	131 (53.7%)
Severe complications (Clavien–Dindo II–IV), *n* (%)	83 (34.0%)
Median hospital stay, days (range)	18 (5–113)
Median pre-operative hospital stay, days (range)	7 (2–44)
Adjuvant therapy, *n* (%)	136 (55.7%)

BMI, body mass index; SD, standard deviation.

In total, 10 patients died within 90 days after surgery and were excluded from the analysis. The post-operative morbidity after surgery was 53.7% (131/244); of the 131 patients, 83 had Clavien–Dindo grade II or higher complications. Major complications (Clavien–Dindo grades II–III) consisted of bile leakage (*n *=* *20), peritoneal cavity infection (*n *=* *11), lung infection (*n *=* *14), sepsis (*n *=* *2), hemorrhage (*n *=* *10), hepatic failure (*n *=* *8), stress ulcer (*n *=* *5), and others (*n *=* *13).

### Overall survival

**Figure 1.**
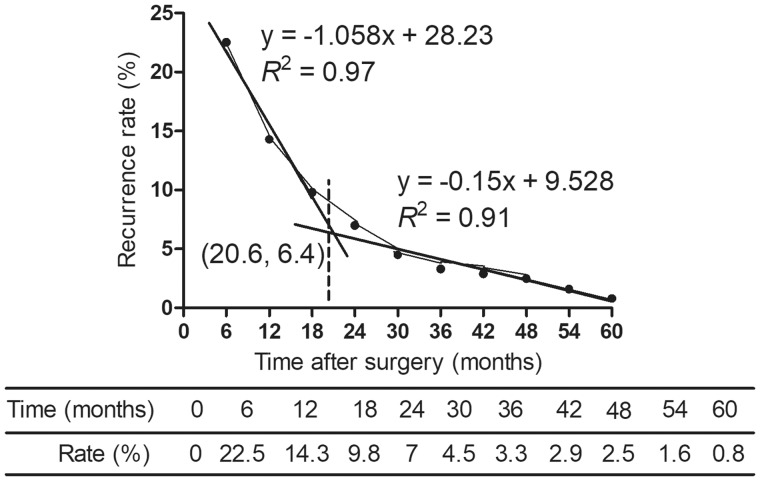
Determination of the optimal cutoff value for early and late recurrence of type IV perihilar cholangiocarcinoma (pCCA). Recurrence was divided into two periods according to the slope of the curves identified with linear regression. The functions of the two straight lines were *y* = –1.058*x* + 28.23 and *y* = –0.15*x* + 9.528, respectively. The intercept point of the two lines was 20.6 months, thus 21 months was defined as the cutoff to distinguish early from late recurrence for type IV pCCA.

**Figure 2.**
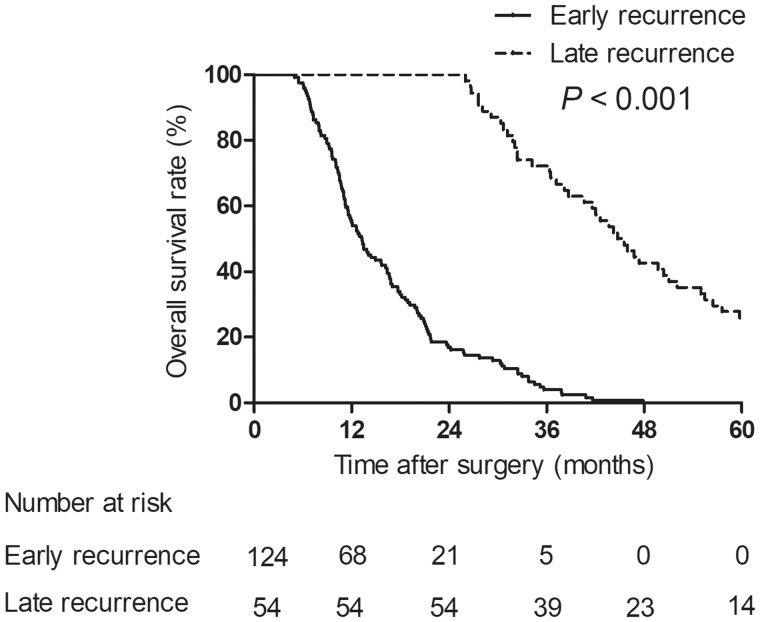
Overall survival rates of patients with type IV pCCA between early- and late-recurrence groups

The median follow-up time for all patients was 26.5 months. The median overall survival time was 25 months and the 1-, 3-, and 5-year overall survival rates were 77.0%, 32.8%, and 16.8%, respectively. Among 244 patients, 178 (73.0%) experienced tumor recurrence after curative resection; of them, 90 (50.6%) experienced recurrence within 1 year, 149 (83.7%) within 3 years and 168 (94.4%) within 5 years. The optimal cutoff point for distinguishing between early and late recurrence was decided based on the recurrence rates calculated every 6 months. Twenty-one months was defined as the cutoff to distinguish early from late recurrence for type IV pCCA (Figure 1). The 1-, 3- 5-year overall survival rates of patients in the early-recurrence group were significantly lower than those of patients in the late-recurrence group (Figure 2).

**Figure 3.**
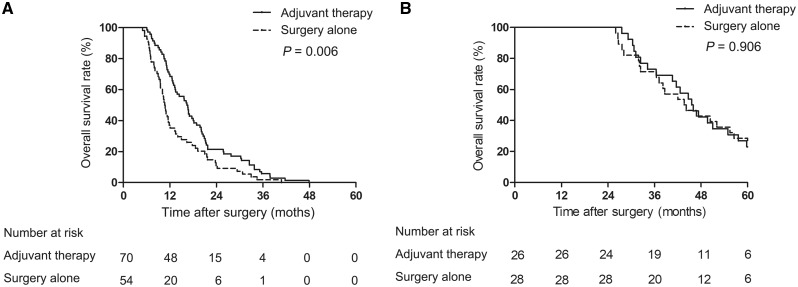
Overall survival rates of patients with type IV pCCA between adjuvant-therapy and surgery-alone groups. (**A**) Early-recurrence subgroup. (**B**) Late-recurrence subgroup.

In the early-recurrence subgroup, the overall survival rate of patients undergoing adjuvant treatment was significantly higher than that of patients undergoing surgery alone (Figure 3A). However, in the late-recurrence subgroup, the overall survival rate was not significantly different between the patients treated with and without adjuvant therapy (Figure 3B).

### Univariate and multivariate analyses of the association of clinicopathological variables with early recurrence

Table 2 shows the relationships between early recurrence and multiple clinicopathological variables. In univariate analysis, increased total bilirubin level, CA19-9 > 200 U/mL, R1 resection margin, higher N category, positive lymphovascular invasion, and tumor size >3 cm were positive predictors of early recurrence. Multivariate analysis using the logistic-regression model demonstrated that CA19-9 level, resection margin, AJCC N stage, and the presence of lymphovascular invasion were independent risk factors associated with early recurrence following curative-intent resection of type IV pCCA.

**Table 2.** Analysis of risk variables for early recurrence (≤21 months) of type IV pCCA after curative surgery

Variable	Univariate analysis			Multivariate analysis	
	Early-recurrence group (*n* = 124)	21-month recurrence-free group (*n* = 120)	*P*-value	HR (95% CI)	*P*-value
Sex			0.209		
Male	83	71			
Female	41	49			
Age (years)			0.317		
<70	108	99			
≥70	16	21			
Total bilirubin (μmol/L)	229.85 ± 189.53	183.23 ± 149.37	0.031	1.001 (0.999–1.002)	0.326
CA19-9 (U/mL)			0.003	1.969 (1.119–3.465)	0.019
<200	43	64			
≥200	81	56			
Albumin (g/L)			0.890		
≤40	91	89			
>40	33	31			
Resection margin			<0.001	2.295 (1.246–4.229)	0.008
R0	70	94			
R1	54	26			
Tumor differentiation			0.090		
Well	24	34			
Moderate	82	77			
Poor	18	9			
AJCC T category			0.100		
T1	5	12			
T2	74	62			
T3	36	42			
T4	9	4			
AJCC N category			0.001	1.831 (1.203–2.789)	0.005
N0	62	88			
N1	43	24			
N2	19	8			
AJCC stage			0.063		
I	4	8			
II	39	49			
III	62	55			
IV	19	8			
Portal-vein encasement			0.108		
Positive	27	37			
Negative	97	83			
Hepatic-artery invasion			0.081		
Positive	18	9			
Negative	106	111			
Lymphovascular invasion			<0.001	3.150 (1.545–6.421)	0.002
Positive	40	14			
Negative	84	106			
Perineural invasion			0.281		
Positive	82	87			
Negative	42	33			
Tumor size			0.024	1.797 (0.835–3.870)	0.134
≤3 cm	96	106			
>3 cm	28	14			

pCCA, perihilar cholangiocarcinoma; HR, hazard ratio; CI, confidential interval; AJCC, American Joint Committee on Cancer.

### Scoring system for predicting early recurrence

On the basis of multivariate logistic-regression analysis, a scoring system for predicting early recurrence was defined (Table 3). Each of the following clinicopathological factors was valued at a score of 1: serum CA19-9 levels of >200 U/mL, AJCC N1 category, R1 resection margin and positive lymphovascular invasion; AJCC N2 stage was valued at a score of 2. The numbers of patients with scores of 0, 1, 2, 3, 4, and 5 were 11, 29, 45, 22, 14, and 3, respectively. Patients with scores of 0, 1, 2, 3, 4, and 5 had early-recurrence rates of 23.9%, 38.7%, 60.0%, 78.6%, 83.4%, and 100%, respectively (*P *<* *0.001).

**Table 3.** Scoring system for predicting early recurrence of type IV pCCA after curative surgery

Factor	Score
CA19-9 (U/mL)	
<200	0
≥200	1
Resection margin	
R0	0
R1	1
AJCC N stage	
N0	0
N1	1
N2	2
Lymphovascular invasion	
Negative	0
Positive	1

pCCA, perihilar cholangiocarcinoma.

**Figure 4.**
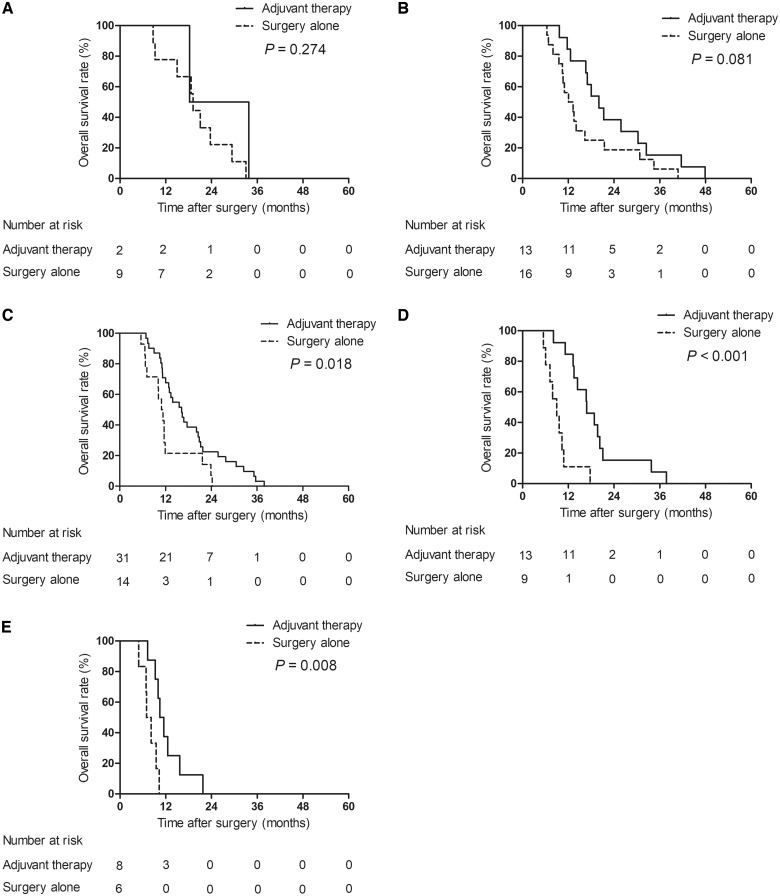
Overall survival rates of type IV pCCA patients with early recurrence between adjuvant-therapy and surgery-alone groups. (**A**) Patients with a score of 0. (**B**) Patients with a score of 1. (**C**) Patients with a score of 2. (**D**) Patients with a score of 3. (**E**) Patients with a score of 4.

Survival analysis for type IV pCCA patients with early recurrence was further performed on the basis of these scores. For the patients with scores of 0–1, the overall survival rate was not significantly different between the patients undergoing adjuvant therapy and those undergoing surgery alone (Figure 4A and B). For patients with scores of 2–4, the overall survival rate was significantly higher in patients receiving post-operative adjuvant therapy than in those receiving radical surgery alone (Figure 4C, D, and E). Survival analysis was not performed in the patients with scores of 5 due to the small sample size.

## Discussion

Radical resection remains the only treatment option for pCCA patients to achieve long-term survival [15–17]. Radical surgery for type IV pCCA is particularly recognized as technically challenging due to the complexity of the intact resection of locally advanced tumors [18]. Thus, a positive resection margin is more common in type IV pCCA than in type I/II and type III tumors [19]. Recurrence after surgery impairs the quality of life and long-term survival of patients [13, 20]. Early recurrence after curative surgery has been reported to be associated with worse prognosis in hepatobiliary carcinoma [9, 12, 21]. Zhang *et al*. [9] defined early recurrence as radiologically diagnosed recurrence within 2.5 years after curative surgery for pCCA patients. We extracted type IV pCCA patients from the database and found that 21 months was the optimal cutoff point to distinguish early and late recurrence of type IV pCCA after radical resection. Compared with patients who had late recurrence, patients with early recurrence had a lower 5-year overall survival rate. We also identified several potentially valuable factors associated with early recurrence, which may help guide decisions regarding post-operative surveillance and adjuvant therapy for patients who are likely to experience early recurrence.

CA19-9 is the most investigated tumor biomarker and has been proven to be a predictor of prognosis in numerous cancers [22, 23]. In our research, patients with CA19-9 >200 U/mL were more likely to experience early recurrence than those with CA19-9 ≤200 U/mL (59.1% vs. 40.2%, *P *=* *0.003). However, two variables that might affect the CA19-9 level were not taken into consideration in our study: first, it has been reported that patients with a Lewis-negative phenotype cannot secrete CA19-9 and were excluded from previous studies [24]; second, to minimize the effect of obstructive jaundice on the CA19-9 level, former cohorts stratified patients into two groups on the basis of hyperbilirubinemia [25]. The two aforementioned variables might affect the CA19-9 level. However, our cohort neither precluded the Lewis-negative population nor stratified patients by bilirubin level, which should be taken into account when explaining the results of our study.

**Figure 5.**
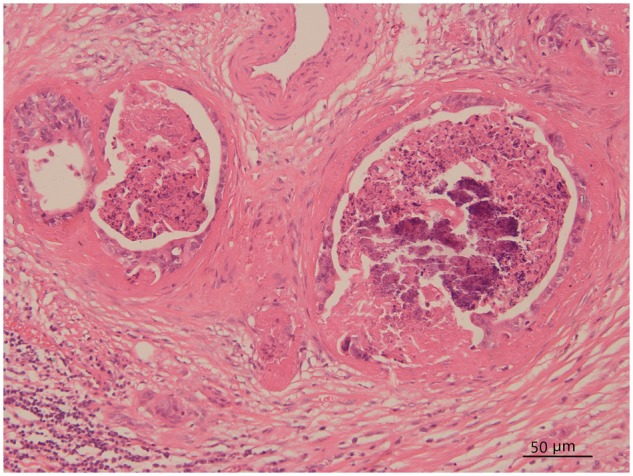
Lymphovascular invasion in a case of type IV pCCA (hematoxylin and eosin staining, original ×200).

Other tumor-related factors strongly significantly associated with early recurrence in our cohort included AJCC N stage category and lymphovascular invasion. A positive relationship between N stage and early recurrence was demonstrated for pCCA in a previous study [9]. While the pathologic classification in previous studies was determined by the 7th edition of the AJCC staging system, in which lymph-node metastases were stratified according to the distribution of lymph nodes, our cohort followed the 8th edition of the AJCC staging system and stratified the N stage category on the basis of the number of positive lymph nodes. In the current study, we similarly found that patients in the early-recurrence group had a more advanced AJCC N stage category than patients in the late-recurrence group (*P *=* *0.001). Lymphovascular invasion has been reported to be significantly strongly associated with poor lower disease-free and overall survival rates for patients with type IV pCCA [26]. In our analysis, lymphovascular invasion was a significant factor associated with early recurrence. According to the AJCC guidelines, lymphovascular invasion refers to tumor involvement in arterial vessels, venules, and lymphatic channels [27]. It has been reported that hematoxylin and eosin (HE) staining along with immunohistochemical staining using D2-40 antibody could improve the detection of lymphovascular invasion [27, 28]. Lymphovascular invasion was confirmed by HE staining alone in our hospital (Figure 5) and thus the presence of lymphovascular invasion may have been underestimated in our study.

Surgery-related factors such as the resection margin could also influence oncologic outcomes [29]. Patients with R0 margins had a decreased risk of early recurrence in our study compared with those with R1 margins. This finding has further stressed the importance of R0 resection to prevent early recurrence; to guarantee R0 resection, measures such as intra-operative frozen-section examination should be routinely performed [30]. Because radical resection for type IV pCCA is considered technically challenging, it was encouraging to find that the R0 resection rate was 67.2% in our study.

A scoring system including CA19-9 level, AJCC N category, lymphovascular invasion, and resection margin was constructed on the basis of multivariate analysis. With increasing scores, the probability of early recurrence for type IV pCCA patients increases. Therefore, for patients with a high score after radical surgery, close post-operative surveillance is necessary for detecting early recurrence. Several studies have investigated the optimal cutoff point for early recurrence of hepatobiliary tumors [9, 12, 31]. However, whether the definition of early recurrence can help guide post-operative therapy remains unclear. During the past decades, many mechanisms underlying tumor progression and therapeutic regimens have been reported, yet the effectiveness of adjuvant therapy for pCCA remains debatable. Recent studies showed that nuclear expression of the cytoskeletal protein S100A4 and the cancer stem cell marker CD133 are practical predictors of tumor progression, as they can be tested in histologic specimens [32, 33]. In particular, Cadamuro *et al*. [34] indicated that nuclear S100A4 is a promising therapeutic target aimed at preventing metastatic dissemination of pCCA. Kim *et al*. [35] reported that radical surgery combined with post-operative adjuvant radiochemotherapy benefited long-term survival in patients with R1 margins and positive lymph nodes. Borghero *et al.* [36] also demonstrated that patients at high risk (with R1 margin or positive lymph nodes) for recurrence treated with radical surgery and radiochemotherapy had a comparable overall survival rate to those (with R0 margin and negative lymph nodes) who underwent surgery alone. The most commonly used chemotherapeutic agents are gemcitabine and 5-FU, which are used as single agents and in combination with other drugs such as leucovorin, cisplatin, and oxaliplatin [37–40]. However, previous studies showed controversial results regarding the effect of chemotherapy on the prognosis of cholangiocarcinoma [41–44]. A prospective study indicated that cisplatin plus gemcitabine is a more effective treatment option than cisplatin/gemcitabine alone for patients with locally advanced biliary cancer [45]. Our results showed that post-operative adjuvant therapy provided a survival benefit for patients with early recurrence, whereas adjuvant therapy did not influence the overall survival of patients with late recurrence. Compared with patients with late recurrence, patients with early recurrence were more likely to be affected by the above risk factors, which may explain why they benefited from adjuvant therapy. We further assumed that patients with more risk factors are more likely to benefit from adjuvant therapy. Thus, patients in the early-recurrence group were stratified based on their scores in the scoring system. Our results showed that, for patients with scores of 2–4, the patients undergoing adjuvant therapy had a significantly higher overall survival rate than those undergoing surgery alone. However, the patients with a score of 0–1 did not significantly benefit from post-operative adjuvant therapy. All patients with a score of 5 underwent post-operative adjuvant therapy. Therefore, type IV pCCA patients with scoring system scores above 2 are recommended to receive adjuvant therapy after curative resection to improve overall survival.

Our scoring system for predicting early recurrence for Bismuth–Corlette type IV perihilar cholangiocarcinoma after curative surgery is simple and inexpensive, and thus it may help in deciding on post-operative-surveillance and adjuvant-therapy strategies, especially for those with multiple risk factors. However, several limitations of the current study should be taken into account. First, as this was a retrospective and uncontrolled cohort collected at a single center, selection bias was inevitable. Second, concomitant factors such as obstructive jaundice and methods of histologic examination may influence the CA19-9 level and detection rates of lymphovascular invasion, which were not sufficiently taken into consideration. Third, our cohort included only three patients with scoring system scores of 5, hence a larger sample size is required to estimate the impact of adjuvant therapy on this subgroup. Finally, our scoring system was applied only in our institution. A multicenter prospective study should be designed to validate and improve our scoring system in the future.

In summary, a simple scoring system including CA19-9 level, AJCC N category, lymphovascular invasion, and resection margin could predict early recurrence for Bismuth–Corlette type IV pCCA after radical surgery.
